# New Plasmids for *Fusarium* Transformation Allowing Positive-Negative Selection and Efficient Cre-*loxP* Mediated Marker Recycling

**DOI:** 10.3389/fmicb.2018.01954

**Published:** 2018-09-11

**Authors:** Krisztian Twaruschek, Pia Spörhase, Herbert Michlmayr, Gerlinde Wiesenberger, Gerhard Adam

**Affiliations:** Department of Applied Genetics and Cell Biology, University of Natural Resources and Life Sciences, Vienna, Austria

**Keywords:** thymidine kinase, negative selectable marker, *Fusarium*, marker recycling, Cre-*loxP*, transformation

## Abstract

In filamentous fungi such as *Fusarium graminearum*, disruption of multiple genes of interest in the same strain (e.g., to test for redundant gene function) is a difficult task due to the limited availability of reliable selection markers. We have created a series of transformation vectors that allow antibiotic-based selection of transformants and subsequent negative selection for marker removal using thymidine kinase fusions combined with the Cre*-loxP* system. The fusion genes contain commonly used C-terminal drug resistance markers, either *nptII* (G418), *nat1* (nourseothricin), or *hph* (hygromycin B). These resistance genes are fused to the sequence encoding *Herpes simplex* virus thymidine kinase (HSVtk). Despite the presence of the 1 kb HSVtk gene (about ∼30% increase in total marker size), there is only a slight reduction in transformation efficiency on a molar basis. The fusion genes expressed under the *Trichoderma* pyruvate kinase (PKI) promoter also confer antibiotic resistance in *Escherichia coli*, allowing straightforward construction of disruption plasmids. For removal of the *loxP* flanked resistance cassettes, protoplasts of transformants are directly treated with purified Cre recombinase protein. Loss of the HSVtk containing cassette is selected by restoration of resistance to 5-fluoro-2-deoxyuridine (FdU). As a proof of principle, we demonstrated the efficiency of the HSVtk-based marker removal in *Fusarium* by reversing the disruption phenotype of the gene responsible for production of the red pigment aurofusarin. We first disrupted the *FgPKS12* gene via integration of the *loxP*-flanked HSVtk*-nptII* cassette into the promoter or the first intron, thereby generating transformants with a white mycelium phenotype. Using Cre recombinase and FdU, the selection marker was subsequently removed, and the resulting transformants regained red pigmentation despite the remaining *loxP* site. We also found that it is possible to remove several unselected *loxP*-flanked cassettes with a single Cre protein treatment, as long as one of them contains a negative selectable HSVtk cassette. The negative selection system can also be used to introduce allele swaps into strains without leaving marker sequences, by first disrupting the gene of interest and then complementing the deletion *in situ* with genomic DNA containing a different allele.

## Introduction

*Fusarium graminearum* is a filamentous plant pathogenic fungus that causes *Fusarium* head blight in wheat, barley and other economically relevant small grain cereals, as well as ear rot in maize. Due to the ubiquitous presence and the broad host range of the pathogen, these diseases, which cause major yield losses and mycotoxin contamination of harvested grains, are a matter of worldwide concern. The most prominent mycotoxin of *F. graminearum* is the type B trichothecene deoxynivalenol (DON), along with several of its derivatives varying in acetylation and hydroxylation patterns. However, besides trichothecenes, *Fusarium* is seemingly capable of producing many other secondary metabolites. Based on the genome sequence, Sieber et al. (2014) predicted 67 gene clusters in *F. graminearum* with significant enrichment of putative secondary metabolism related enzymatic functions. Elucidation of the metabolites corresponding to these clusters might give further insight into the virulence mechanisms of the pathogen. However, knockouts of individual secondary metabolite biosynthesis genes can have little impact on virulence (Gaffoor et al., 2005), and multiple gene deletions might be required to reveal redundancies. For instance, in *F. graminearum*, disruption of the genes necessary for production of the three different siderophores malonichrome (*NPS1*), ferricrocin (*NPS2*), and triacetylfusarinine (*NPS6*) led to drastically reduced virulence (Oide et al., 2014).

The toolbox available for the genetic engineering of *Fusarium* is limited to a small number of reliable selectable marker genes and affordable selective agents. The most commonly used resistance genes are *hph* (hygromycin, hyg), *nptII* (G418, kan), and *nat1* (nourseothricin, nat). While these selectable markers enable gene knockouts and replacements, they cannot be efficiently recycled to produce multiple mutations in one strain. To our knowledge, consecutive gene deletions in *Fusarium* with more than three genes have not been published. For other fungi, however, several marker recycling protocols have been described. Such methods commonly involve excision of the marker DNA by recombinases, e.g., with the Cre-*loxP* system. In some species, such as baker’s yeast (Sauer, 1987) or *Cryptococcus neoformans* (Patel et al., 2010), the expression of Cre recombinase alone is sufficient to obtain marker-free transformants with reasonable screening effort. Similar results were obtained in *Ashbya gossypii*, where two consecutive gene deletions were done after the first marker had been rescued (Aguiar et al., 2014). As an alternative to Cre, the FLP/FRT system was employed in *Ustilago maydis* for a total of five rounds of sequential gene deletions using a single resistance gene (Khrunyk et al., 2010). The FLP/FRT system was also utilized to remove markers from *Penicillium chrysogenum* and *Sordaria macrospora* (Kopke et al., 2010).

We opted to use the Cre*-loxP* recombination system (Sternberg and Hamilton, 1981) in our constructs to enhance the probability of obtaining the desired recombination event in *Fusarium* protoplasts. Cre recombinase is an enzyme capable of site-specific recombination between two 34-bp *loxP* sites. *LoxP* sites with the same orientation lead to excision of the flanked sequence (Kühn and Torres, 2002). Cre*-loxP* mediated removal of marker cassettes has been used in many organisms, ranging from mammalian systems to filamentous fungi such as *Trichoderma reesei* (Steiger et al., 2011), as well as multiple *Aspergillus* species (e.g., Mizutani et al., 2012; Zhang et al., 2016). The method used most frequently involves inducible expression of Cre recombinase in the target organism. However, it has been reported that direct introduction of Cre is also possible in filamentous fungi by treating protoplasts with the purified protein (Mizutani et al., 2012).

While the use of Cre is sufficient to remove a marker from some organisms, in *F. graminearum* Cre mediated marker loss is possible, but requires extensive screening. Therefore, to reduce screening efforts, negative selectable markers are needed. A method using the counterselectable *amdS* marker combined with 5-fluoroacetamide was published for *T. reesei* (Steiger et al., 2011), however, we found that this method does not work in *F. graminearum*. Forment et al. (2006) used the orotidine-5-phosphate decarboxylase gene (*URA3*/*pyrG*) in *Aspergillus nidulans* for counterseletion with 5-fluoroorotic acid, as did Royer et al. (1999) in *F. venenatum*. A paper addressing the issue of multiple integrations in *Aspergillus* was published using only two auxotrophy markers *AfpyroA* (pyridoxine) and *AfriboB* (riboflavin), although this method could also possibly be adapted for a pair of resistance markers such as *nptII/hph*. By replacing one marker with the other over multiple rounds of transformations, several heterologous secondary metabolism-related genes could be expressed in one locus in *A. nidulans*. Furthermore, by using the previously mentioned *pyrG* dual-selectable marker, marker recycling was achieved using 5-fluoroorotic acid counterselection (Chiang et al., 2013). The downside of these methods is that they require auxotrophic strains, which might interfere with virulence.

*Herpes simplex* virus thymidine kinase (HSVtk) can be used as a conditional negative selectable marker by supplementing the nucleoside analog 5-fluoro-2-deoxyuridine (FdU) to the culture medium. Cells expressing HSVtk can convert FdU to FdU-monophosphate (FdUMP), which, when incorporated into DNA and RNA, leads to DNA fragmentation upon excision-repair, and functional impairment of tRNA, mRNA and rRNA-related processes. Furthermore, FdUMP forms a stable complex with thymidylate synthase, thus blocking thymidine synthesis (Daher et al., 1990). HSVtk negative selection was initially developed in mammalian cell lines, in which non-homologous end joining mechanisms strongly compete with homologous recombination-based integration of foreign DNA. Using the original HSVtk protocol, the number of incorrect ectopic mutants could be reduced by placing HSVtk on the flanks of a linearized homologous transformation cassette (Müller, 1999). The method was adapted for fungi such as *Leptosphaeria maculans* (Gardiner and Howlett, 2004) and also *Fusarium oxysporum* (Khang et al., 2005). Single-gene fusions of HSVtk and resistance genes have been described using either the hygromycin phosphotransferase (*hph*) gene in N-terminal position (Lupton et al., 1991), or the neomycin phosphotransferase (*npt*) gene in C-terminal position (Schwartz et al., 1991). HSVtk-fusion genes were proposed as a general strategy by Karreman (2000). An N-terminal bleomycin resistance/C-terminal HSVtk-fusion gene was used for an allele swap of the veA locus of *Aspergillus fumigatus*, requiring only a limited screening effort (Krappmann et al., 2005), and a series of fused positive/negative selectable markers was recently developed for fission yeast by Amelina et al. (2016). However, placing the antibiotic resistance gene at the N-terminus increases the risk of obtaining transformants that contain a truncated fusion gene, which confers antibiotic resistance but lacks the counterselectable marker.

The aim of our study was to develop vectors allowing multiple gene disruptions in one strain. For this purpose, we generated three fusion genes comprising HSVtk at the N-terminus and one of the following three resistance genes at the C terminus: neomycin phosphotransferase II (*nptII*), nourseothricin acetyltransferase (*nat1*), or hygromycin B phosphotransferase (*hph*). We tested whether genes can be disrupted and the marker removed from the genome by counterselection of the HSVtk gene. We could show that the marker can be removed either by homologous recombination, or by using the Cre*-loxP* system. The developed vectors are useful tools for gene editing in *Fusarium*, and probably also for other ascomycete fungi.

## Materials and Methods

### Strains, Media, and Conditions

*Fusarium graminearum* PH-1 (NRRL 31084, FGSC 9075) was used as the host strain for transformations. It was grown at 20°C on Fusarium minimal medium (FMM) containing 1 g/L KH_2_PO_4_, 0.5 g/L MgSO_4_.7H_2_O, 0.5 g/L KCl, 2 g/L NaNO_3_, 30 g/L sucrose, and 20 g/L agar, as well as 200 μL/L of a trace element solution that was added after autoclaving. One hundred milliliter of trace element solution was prepared by adding 5 g citric acid, 5 g ZnSO_4_.6H_2_O, 1 g Fe(NH_4_)_2_(SO_4_)_2_.6H_2_O, 250 mg CuSO_4_.5H_2_O, 50 mg MnSO_4_, 50 mg H_3_BO_4_, and 50 mg Na_2_MoO_4_.2H_2_O to deionized water (Leslie and Summerell, 2008). Transformants expressing either of the *hph, nat1*, or *nptII* resistance genes were cultivated on FMM agar with added selection agents in the following concentrations: 100 mg/L hygromycin B (Carl Roth #CP13.3), 25 mg/L nourseothricin dihydrogen sulfate (Werner BioAgents), or 30 mg/L G418 (Fermtech Garching, Germany), respectively. To counterselect transformants expressing HSVtk, 12.3 μg/L FdU (=50 nM; Sigma-Aldrich #F0503) was added to FMM agar. Conidia of *F. graminearum* were generated by inoculating 50 mL of mung bean extract (MBS, filtrate of 10 g mung beans per L water boiled for 20 min) in a 250 mL baffled flask with fungal mycelium. After 3 days of incubation on a shaker at 140 rpm at 20°C in the dark, conidia were obtained by removing mycelium using sterilized glass wool and subsequent sedimentation over night at 4°C.

For side-by-side testing of *Fusarium PKS12* phenotypes, nine agar plugs with a defined diameter were punched from 7-day-old FMM plate cultures using a cork borer (size 1, ∼4 mm inner diameter), and 1-mm slices with mycelium were placed on a 94 mm FMM agar plate in a 3 × 3 grid using a scalpel.

*Escherichia coli* strains DH10B and BL21 were employed for standard cloning procedures and for expression of Cre recombinase, respectively. The following media were used for *E. coli* cultivation: LB (5 g/L yeast extract, 10 g/L tryptone, 10 g/L NaCl, adjusted to pH 7) and TB (12 g/L tryptone, 24 g/L yeast extract, 4 mL/L glycerol, 23.14 g/L KH_2_PO_4_ and 125.41 g/L K_2_HPO_4_), 100 mg/L ampicillin were added when required.

### PCR Screening

For PCR screening of fungal transformants, standard 20-μL PCR reactions were used (Sambrook and Russell, 2001). Homemade *Taq* DNA polymerase was used (Pluthero, 1993). Parameters for routine PCRs were as follows: 2.5 mM MgCl_2_, 20 mM Tris-Cl pH 8.4, 50 mM KCl, 0.2 mM dNTPs each, 1 μM primers each. PCR cycler parameters: denaturation (95°C) and annealing both 30 s, elongation 72°C for 1 min/kb, 30 cycles, initial denaturation: 2 min/95°C, final elongation: 5 min/72°C (10 min for > 1 kb). For annealing temperatures of each primer pair used, refer to **Supplementary Table S1**. Templates for PCR were prepared from small amounts of hyphae, which were transferred into 50 μL Tris-EDTA (TE pH 7.5) buffer using a sterile toothpick and were then microwaved for 45 s at 800 W. From these extracts, 2-μL aliquots were added to 20-μL PCR reactions. For the primers used and the expected PCR band lengths, refer to **Supplementary Table S1**.

### Construction of Plasmids

#### General

Plasmid DNA was produced in *E. coli* strain DH10B using standard procedures (Sambrook and Russell, 2001). Primers used for cloning are listed in **Supplementary Table S2**. Q5 DNA polymerase was used for cloning purposes (New England Biolabs, Ipswitch, MA, United States). PCR protocol and mixture was used as stated in the manufacturer’s instructions. T_m_ values for each primer pair were calculated using the manufacturer’s online T_m_ calculator tool^1^. For gene knockouts in *F. graminearum*, the HSVtk-fusion cassettes were flanked by homologous untranslated regions of approximately 500 bp using the Gibson assembly method (Gibson et al., 2009). Refer to **Supplementary Table S3** for a list containing all plasmids in this study, and **Supplementary Table S4** for flanking region lengths.

#### Construction of the HSVtk Test Construct pKT235

To test the feasibility of HSVtk-FdU counterselection in *F. graminearum* strain PH-1, the HSVtk gene (sequence originally published by McKnight, 1980) was PCR-amplified from plasmid pCGS966 (Smith et al., 1990) using primers with added XbaI and KpnI restriction sites (#3099 and #3100) and ligated to the fungal expression vector pAB86, yielding pKT235. pAB86 is derived from pRLMex30 (Mach et al., 1994) containing an additional *A. nidulans* gpdA promoter. In pAB86, a gene of interest can be cloned downstream of the strong constitutive *T. reesei* pyruvate kinase promoter (GenBank ID CP016233.1, bases 1063628–1064369). The XbaI and KpnI cloning sites lie upstream of the hygromycin resistance cassette containing the *hph* gene under control of the *A. nidulans* constitutive glyceraldehyde-3-phosphate dehydrogenase promoter (*gpdA;* GenBank ID BN001302.1, bases 576408–577607). As a single terminator downstream of *hph*, the *T. reesei* cellobiose hydrolase II (*CBH2*) terminator was used (GenBank ID CP016238.1, bases 3804665–3805218). The HSVtk ORF was confirmed by sequencing. All clones obtained from the template contained a silent G102A mutation in comparison to the published HSV-1 strain 17 genome (GenBank JN555585.1, bases 47803–46676), but another published human *Herpes* virus 1 genome (GenBank X14112.1, bases 2678–3803) also has this mutation. A second silent mutation, T969C, was found in the amplified HSVtk coding sequence, which is present in the HSV-1 strain 17 sequence (JN555585), but absent from the X14112.1 sequence. In conclusion, both nucleotide changes exist in sequenced *Herpes* virus genomes and are unlikely to affect translation efficiency.

#### Construction of the HSVtk-*nptII* Containing Vector pKT245

The HSVtk-*nptII* gene was constructed via fusion PCR (Horton, 1997). Individual HSVtk and *nptII* fusion fragments were PCR-amplified from the plasmids pCGS966 (Smith et al., 1990) and pII99 (Namiki et al., 2001), respectively, using fusion primers (#3099 and #3157; #3158 and #3159) with added restriction sites and overhangs. The resulting 1.2 kb and 0.8 kb bands were subjected to a fusion PCR (primers #3099 and #3157), yielding a 2.0 kb HSVtk-*nptII* fragment. The fusion gene was digested with XbaI and KpnI and ligated to the 6.6 kb pAB86 backbone to yield plasmid pKT241. The fusion gene was verified by sequencing.

The 3.6 kb BbsI-AsiSI fragment containing the HSVtk-*nptII* resistance cassette was ligated into a *loxP*-containing backbone (4.6 kb) obtained by digestion of pTS101 with BbsI and AsiSI (pTS101 originates from pASB43 without *amdS*, see **Supplementary Figure S1**). The resulting plasmid pKT244 contained the hygromycin resistance gene *hph* under control of the gpdA promoter downstream of the HSVtk-*nptII* cassette. To generate a HSVtk-*nptII* plasmid without the hygromycin cassette, pKT244 was digested with EcoRI, treated with Klenow polymerase, and subsequently digested with SpeI. This generated the 5.2 kb backbone containing one *loxP* site upstream of the HSVtk-*nptII* gene, but no terminator. The terminator and the second *loxP* site were provided by a 0.8 kb NsiI/Klenow and EcoRI-fragment from pTS101. The resulting plasmid, pKT245, contained the following construct: *loxP*-*PKI* promoter-HSVtk-*nptII*-*CBH2* terminator-*loxP*.

#### Construction of the HSVtk-*nat1* Containing Vector pKT247

The 0.6 kb *nat1* gene was obtained from pNR1 (Malonek et al., 2004) by digestion with BamHI, generation of blunt ends via Klenow fragment and subsequent EcoRI digest. The 7.4 kb backbone was isolated by BsmI digest of pKT244, Klenow treatment, and EcoRI digest. The *nat1* fragment was ligated to the backbone to produce pKT246. Using this strategy, seven bases from the 3′ end of the *oliC* promoter were appended to the first five bases of the pKT241 HSVtk*-nptII* linker without disrupting the open reading frame. The hygromycin resistance cassette was removed from pKT246 by EcoRI digestion followed by Klenow fill-in and SpeI digest (5 kb). pTS101 was digested with NsiI, blunted with Klenow fragment, and digested by SpeI (0.8 kb). Insert and backbone were ligated to construct the final HSVtk*-nat1* plasmid pKT247.

#### Construction of the HSVtk-*hph* Containing Vector pKT248

*Hph* with added BsmI and KpnI restriction sites was PCR-amplified from pAB86 using primers #3844 and #3845, digested with BsmI and KpnI and ligated to the 5.2 kb pKT245 backbone to yield pKT248. The ORF was verified by sequencing. Compared to the original *hph* sequence (Gritz and Davies, 1983/GenBank ID K01193.1, bases 210–1236), four silent mutations are present, which were also present in the template plasmid (T30C, A246G, T381C, T759C).

#### Fusion Protein Linker Modifications (pKT292-*nptII* and pKT293-*hph*)

The original 9-bp linker sequence of the HSVtk*-nptII* and HSVtk*-hph* constructs was exchanged for linkers of two lengths: short linker (SL) and long linker (LL). The SL and LL versions of HSVtk*-nptII* and HSVtk*-hph* were constructed by ligation of an NsiI-compatible homologous pair of hybridized ssDNA oligonucleotides into the NsiI site (SL: #4228 and #4229, LL: #4230 and #4231). Hybridization of ssDNA to produce sticky-ended dsDNA fragments was done by incubating two primers (10 μM of each oligonucleotide) in one tube at 95°C in 1X PCR buffer for 5 min and slowly cooling the tubes to room temperature. One microliter of each mix was added to a ligation reaction containing 50 ng of the NsiI-digested backbones of either pKT245 (*nptII*) or pKT248 (*hph*). Six candidates of each transformant were transferred to agar plates containing reduced amounts of the respective antibiotic (17.5 mg/L kanamycin or 37.5 mg/L hygromycin) in addition to non-resistant controls. The LL candidates capable of forming single colonies were further verified by PCR for correct orientation of the linker and designated pKT292 (*nptII*) and pKT293 (*hph*).

#### Addition of New Flanking Polylinker Sequences

To facilitate digestion/ligation cloning of the HSVtk constructs for future applications, a custom designed polylinker (multiple cloning site) was ordered as synthetic DNA cloned in the Eurofins standard ampicillin vector pEX-A2 (Eurofins Genomics GmbH). The plasmid was cleaved with NotI, and the 0.1 kb NotI-digested polylinker was ligated to the 2.4 kb NotI backbone of pKT245, resulting in the vector pKT290. pKT292 (*nptII*), pKT247 (*nat1*), and pKT293 (*hph*) were cleaved with SalI and SpeI, the resulting 3.3–3.5 kb fragments contained the resistance marker cassettes flanked by *loxP* sites. Each of these fragments was ligated to the SalI and SpeI digested pKT290 backbone, thereby generating the plasmids pKT300 (*nptII*), pKT301 (*nat1*), and pKT302 (*hph*). For construction of plasmids lacking *loxP*, pKT292, pKT247, and pKT293 were digested with XhoI. The resulting 3.3–3.5 kb fragments containing the resistance marker cassettes without *loxP* sites were ligated to XhoI-cleaved pKT290. Two orientations of each plasmid were obtained: pKT303 (*nptII*), pKT304 (*nat1*), and pKT305 (*hph*), in which the resistance cassette has the same orientation as in the *loxP*-flanked variants described above, and pKT311 (*nptII*), pKT312 (*nat1*), and pKT313 (*hph*), in which the resistance cassette was integrated in reverse orientation.

#### Construction of a Plasmid for Production of Cre Recombinase

The ORF coding for Cre recombinase (GenBank accession no. X03453.1) was amplified (from an *E. coli* strain containing a lysogenic phage P1) without a stop codon using primers with added NdeI and XhoI sites (#5089 and #5090). The 1 kb PCR product was digested with NdeI and XhoI and ligated to the pET-21a(+) backbone (Novagen Inc.) cleaved with the same enzymes. The resulting vector contains the Cre recombinase with a C-terminal 6x His tag for purification.

### Purification of Cre Recombinase

*Escherichia coli* BL21 carrying the Cre-pET21a(+) plasmid was inoculated in 10 mL LB+ampicillin and incubated at 37°C overnight. The overnight culture was added to 1 L TB+ampicillin medium to dilute the cells 1:100 for an OD_600_ of approximately 0.1. The culture was incubated at 37°C until reaching an OD_600_ of 1, at which time point lactose was added to a final concentration of 5 g/L for induction of Cre expression. After induction, the cells were further incubated at 25°C and harvested after 16 h by centrifugation (6,000 *g* for 20 min), and disrupted in a French press at 8.3 MPa in three cycles. Cre recombinase was isolated from the protein extracts via immobilized metal ion affinity chromatography (IMAC) using Ni-charged chelating Sepharose (GE Healthcare, Vienna, Austria) following the supplier’s instructions. The buffer was changed to 25 mM phosphate buffer pH 6.5 with a HiPrep 26/10 desalting column (GE Healthcare, Vienna, Austria). A second purification step was cation exchange with Resource S 1 mL columns (GE Healthcare, Vienna, Austria). The protein was bound to the column in 25 mM phosphate buffer pH 6.5 and eluted by applying a linear gradient to 1 M NaCl in 15 column volumes (CV). At this stage, DNase activity could still be detected in the purified protein, therefore, a third purification step was applied. Size exclusion chromatography on a Superdex 75 10/300 column (GE Healthcare, Vienna, Austria) was carried out with a flowrate of 0.5 mL/min with 30 mM Tris-Cl pH 8 and 500 mM NaCl. An equal volume of glycerol was added to the purified Cre recombinase (resulting in 15 mM Tris-Cl pH 8, 250 mM NaCl and 50% glycerol), after which the protein was aliquoted and stored at -20°C. Enzyme activity was assessed through determining the circularization rate of linearized plasmid DNA [pLox2a(+), New England Biolabs] by transforming *E. coli* DH10B with *in vitro* circularization reactions performed with serial dilutions of enzyme preparations. Activity of homemade Cre preparations were compared to that of the commercially available Cre recombinase (New England Biolabs #M0298S).

### Protoplast Transformation

For *Fusarium* transformation and marker pop-out we used the following protocol, which is based on the method published by (Gaffoor et al., 2005). To generate freshly germinated mycelium, 5 × 10^6^ *Fusarium* conidia were used to inoculate 100 mL YEPD medium (0.3% yeast extract, 1% peptone from meat, 2% glucose added from a sterile stock after autoclaving) and shaken overnight at 30°C. Mycelium was harvested by using a sterile Buchner funnel and resuspended in 20 mL protoplasting solution consisting of: 25 g/L Driselase from *Basidiomycetes sp.* (Sigma-Aldrich #D9515), 50 mg/L chitinase from *Streptomyces griseus* (Sigma-Aldrich #C6137), and 5 g/L lysing enzymes from *Trichoderma harzianum* (Sigma-Aldrich #L1412) in 1.2 M KCl (filter sterilized). After gentle shaking at 30°C for 1–3 h, protoplasts were separated from remaining mycelium by filtration using a 30 μm Celltrics filter membrane (Partec GmbH, Germany) into a 50-mL tube and stored on ice. Protoplasts were collected by centrifugation at 1000 *g*, washed with 10 mL STC buffer (1.2 M Sorbitol, 10 mM Tris pH 7.5, 50 mM CaCl_2_) and resuspended in 1 mL STC. Final protoplast concentration was adjusted to 10^8^ per mL, 100 μL of this was added to each transformation reaction. One hundred microliter STC and 50 μL PEG-solution (30% PEG-8000, 10 mM Tris-Cl pH 7.5, 0.5 mM CaCl_2_) were added to each reaction, as well as 10 μg transforming DNA. Transformation reactions were swirled gently and incubated for 20 min, after which 2 mL of PEG-solution was added. After 5 min of further incubation, 4 mL STC was added to complete the transformation mixture.

Six hundred microliter of transformation mixture was added to 15 mL regeneration medium [10 g/L agarose, 1 g/L yeast extract, 1 g/L casein enzymatic hydrolysate (NZ-Amine), 275 g/L sucrose]. The regeneration medium was equilibrated in a 50°C water bath. Usually, 10 plates per plasmid construct were prepared. After 2 h of regeneration for G418, hygromycin B and FdU, and 3 h for nourseothricin, each plate was overlaid with 15 mL regeneration medium containing the respective selective agent (60 mg/L G418 for *nptII*, 100 mg/L nourseothricin for *nat1*, 200 mg/L hygromycin B for *hph*, 123 μg/L FdU).

Transformation plates were incubated in the dark for 4–7 days. Candidates were transferred to selective FMM plates (30 mg/L G418, 50 mg/L nourseothricin, 100 mg/L hygromycin B, 12.3 μg/L FdU) and grown for 4–10 days. Following PCR confirmation of correct DNA integration, transformants were sporulated in liquid MBS and plated onto selective FMM agar to yield single-spore colonies. A single colony was transferred to a fresh selective FMM plate. To ensure that candidates were homokaryotic and no longer contained untransformed nuclei, the process of sporulation and single conidia isolation was repeated to generate “second-generation” transformants.

### Marker Removal Protocol

To remove a *loxP*-flanked marker from an engineered *F. graminearum* strain, the transformation protocol above was followed up to the point of adding transforming DNA to protoplasts. Instead of DNA, 5 μL of homemade Cre recombinase (approximately 5000 units) was added to the transformation mixture. All other steps of the transformation remained unchanged, except for the use of 123 μg/L FdU in the overlay medium.

### *Escherichia coli* Resistance Testing

Overnight cultures of *E. coli* DH10B carrying each of the tested resistance cassettes as well as an untransformed control were diluted to 0.15 OD_600_ and incubated until reaching a cell density of 0.8 OD_600_. Serial dilutions (1:10) were prepared up to D6 (1:1,000,000). One hundred microliter of dilutions 3–5 (*nptII*) and 4–6 (*nat1, hph*) were plated in duplicate on LB agar containing the different concentrations of the required selection agents, as well as on LB+ampicillin (100 mg/L) for controls. The plates were incubated overnight.

### Construction of Vectors for *Fusarium* Transformation

#### Construction of Deletion Vectors for Efficiency Testing and HSVtk-Less Controls

The plasmids pKT245, pKT247, and pKT248 were modified to knock out a number of genes in *F. graminearum* PH-1, resulting in the plasmids pPS45, pPS48, and pPS51, the construction of which is described below (see also **Supplementary Figure S1**). HSVtk-less derivatives were also constructed, as indicated by the “HL” suffix.

##### Construction of the HSVtk-nptII containing disruption vector pPS45

The dual-selectable *T. reesei amdS-hph* cassette was moved from pMS-HALS (Steiger et al., 2011, genotype: *ampR, loxP*-*amdS*-*hph*-*loxP, sacB*, pUC *ori*) to the pUni51 backbone (GenBank accession no. AY260846.1, genotype: *kanR, loxP*, R6Kγ *ori* – requires a *Pir1* host strain) by Cre-mediated recombination, and isolated by selection on kanamycin-containing medium. The plasmid was named pASB2 (genotype: *kanR, loxP-amdS-hph-loxP*, R6Kγ *ori*). In the next step, pUG6 (Gueldener et al., 2002, GenBank accession no. AF298793.1, genotype: *ampR, loxP-kanR-loxP*, pUC *ori*) was transformed in the Cre expressing strain DH10B P1cam in order to lose the fungal kanamycin resistance. The resulting ampR plasmid, named pUG6woKAN, was isolated and transformed in *E. coli* DH10B for DNA preparation. The *loxP-amdS-hph-loxP* cassette was transferred from pASB2 to pUG6woKAN by *in vitro* Cre recombination and subsequent transformation. Transformants carrying the desired construct (genotype: ampR, *loxP-amdS-hph-loxP*, pUC ori) were selected on ampicillin and hygromycin containing media, the resulting plasmid was named pASB43. A 500 bp fragment of the FGSG_02279 5′ UTR was amplified from *F. graminearum* genomic DNA using primers #2783 and #2784. For amplification of the 3′ UTR, primer pair #2785 and #2786 was used, the resulting fragment contained 500 bp of the 3′ UTR directly adjacent to the stop codon. The 5′ UTR was digested with SfiI and SpeI, and ligated to SfiI, SpeI cleaved pASB43 to yield the plasmid pHE31. The 3′ UTR was digested with SalI and SplI, and ligated into pHE31 to generate pHE49. Finally, the *amdS-hph* selection marker in pHE49 was replaced by the 3.5 kb HSVtk-nptII cassette from pKT245 via SalI and SpeI digest and subsequent ligation, yielding pPS45.

##### Construction of the HSVtk-less nptII containing disruption vector pPS45HL

pPS45 was digested with XbaI and NsiI, treated with Klenow fragment and self-ligated to yield an HSVtk-less control plasmid. Despite presence of a second XbaI site in pPS45, no partial digestion was necessary due to its dam methylation by *E. coli* DH10B.

##### Construction of the HSVtk-nat1 containing disruption vector pPS48

The 5′ and 3′ UTRs of the FGSG_03278 gene were amplified using primer pairs #2827 and #2828 and #2829 and #2830, respectively, and cloned into pASB43 in two steps, using SfiI and SpeI to clone the 5′ UTR and SalI and Hind III for the 3′ UTR; the resulting plasmid was named pPS19. pPS48 was constructed by replacing the *hph*-*amdS* cassette in pPS19 by the 3.5 kb HSVtk-*nat1* resistance gene using SalI and SpeI restriction.

##### Construction of the HSVtk-less nat1 containing disruption vector pPS48HL

HSVtk was removed from pPS48 by digestion with XbaI and BamHI, Klenow treatment and self-ligation.

##### Construction of the HSVtk-hph containing disruption vector pPS51

pPS51 contains HSVtk-*hph* from pKT248 between the 5′ and 3′ UTRs of FGSG_00348. It was constructed via Gibson assembly using the PCR products of primer pairs #3946 and #3947 for the 5′ UTR and #3948 and #3949 for the 3′ UTR and a 3.8 kb SalI-SpeI fragment containing HSVtk-*hph* as well as the SalI-NdeI digested pUC19 backbone.

##### Construction of the HSVtk-less hph containing disruption vector pPS51HL (pPS54)

pPS51 was digested with XmaI and XbaI, treated with Klenow fragment and self-ligated to yield the HSVtk-less control plasmid.

#### Construction of *PKS12* Disruption Vectors

For reversible *PKS12* (FGSG_02324) disruption, the HSVtk-*nptII* cassette was integrated at two different loci: between promoter and gene (pKT257), and into the first intron (pKT258).

##### Construction of the reversible PKS12 disruption vector pKT257 (promoter integration)

The *PKS12* promoter insertion vector was assembled using Gibson assembly, combining two restriction fragments from the pKT245 backbone and the pUC19 ori (3.5 kb pKT245 HindIII, SpeI fragment and the 2.5 kb pUC19 SalI, NdeI backbone) with two PCR fragments containing *Fusarium* homology regions amplified from *F. graminearum* PH-1 genomic DNA using primer pairs #3969 and #3970; #3971 and #3972.

##### Construction of the reversible PKS12 disruption vector pKT258 (intron integration)

pKT258 was designed to integrate HSVtk*-nptII* into the first intron, which begins 288 bp downstream of the *PKS12* start codon. The marker cassette was placed between the GT splice donor site and the YNCURAY branch site consensus (Kupfer et al., 2004). The plasmid was constructed in the same way as pKT257 (see above), except using PCR fragments amplified from *F. graminearum* PH-1 genomic DNA using the primer pairs #3969 and #3966; #3967 and #3972.

#### Construction of pPS50, Used to Test Marker Recycling

pPS50 was generated using the HSVtk-*hph* cassette from pKT248 placed between the 5′ and 3′ UTRs of FGSG_16976. It was constructed via Gibson assembly using the PCR products of primer pairs #3942 and #3943 for the 5′ UTR, #3944 and #3945 for the 3′ UTR and a 3.8 kb SalI-SpeI fragment containing HSVtk-*hph* as well as the SalI-NdeI digested pUC19 backbone.

#### Construction of Vectors for Allele Swap of *TRI8*

For generation of an allele-swapped *Fusarium* strain the following vectors were constructed: the *TRI8* deletion construct pKT249 and the seamless 3-ADON *TRI8* swap construct pKT299.

##### Construction of the HSVtk-nat1 based TRI8 disruption vector pKT249

pKT249 was constructed by combining four DNA fragments via Gibson assembly: two PCR-amplified flanking regions (primers #3559 and #3573; #3561 and #3574), the HSVtk*-nat1* cassette on a 3.3 kb XhoI fragment from pKT247 and the SalI and NdeI digested backbone of pUC19.

##### Construction of marker-free 3-ADON TRI8 transformation vector pKT299

pKT299 was generated by Gibson assembly of the 3-ADON *TRI8* PCR fragment (primers #4769 and #4770, 1.3 kb), the 3′ UTR (#4771 and #3574, 0.9 kb), and the AdeI, PstI-digested pKT249 backbone (3.0 kb). As PCR template for 3-ADON *TRI8*, the pCS26 plasmid was used, which contains the 3-acetyl-DON producer allele of *TRI8* obtained from *F. graminearum* strain DSMO4258 (Altpeter and Posselt, 1994; sequence identical to GenBank accession KU572434.1 bases 1875–3173). The 3′ UTR was amplified from *F. graminearum* PH-1 genomic DNA.

## Results

### Construction of Plasmids

**FIGURE 1:**
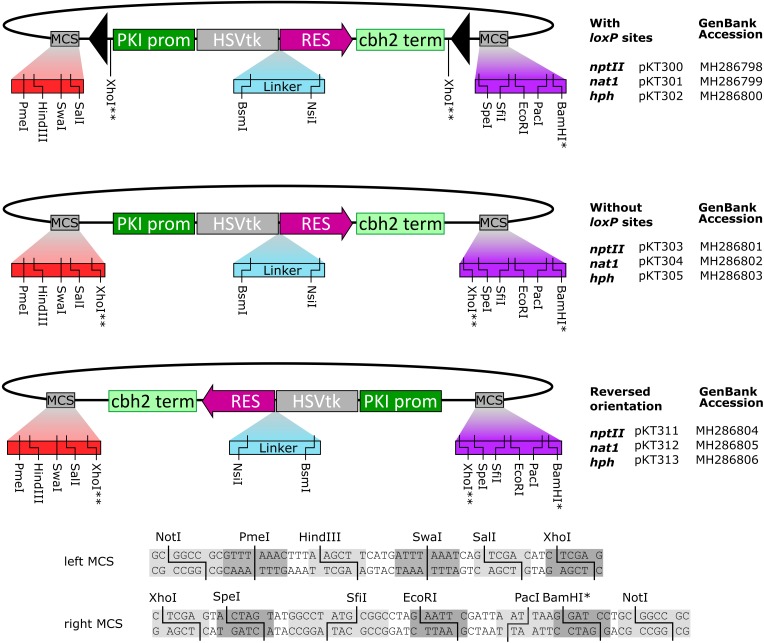
The series of fungal transformation vectors based on HSVtk fusion genes produced in this study. Sequences of the up- and downstream multiple cloning sites (MCS) are shown below. (^∗^) The BamHI site is not unique in the *nat1* constructs. (^∗∗^) XhoI is not unique; two XhoI sites are present, flanking the marker. Full sequences of the constructs are available at GenBank via their respective accession numbers.

We constructed a series of fungal transformation vectors consisting of an N-terminal HSVtk gene fused to one of three different resistance markers: *nptII* (G418), *nat1* (nourseothricin) and *hph* (hygromycin). The cassettes contain the *T. reesei* pyruvate kinase promoter and the cellobiohydrolase II (*CBH2*) terminator, as implemented in its precursor pRLMex30 (Mach et al., 1994). Additionally, custom polylinkers with unique sites were added on both sides of the resistance cassette to facilitate digestion/ligation cloning of flanking regions used for fungal gene disruption. For each resistance gene, *loxP*-flanked variants were generated in addition to vectors without *loxP* sites. For the vectors lacking *loxP* sites, the resistance cassettes were inserted in both orientations relative to the polylinker sites, yielding a total of nine constructs as shown in **Figure 1**. Sequences of the plasmids are available on GenBank (Accession numbers MH286798–MH286806, as shown in **Figure 1**).

### HSVtk-Based Counterselection Using FdU in *Fusarium graminearum*

To determine the functionality of HSVtk and FdU counterselection in *F. graminearum* PH-1, we first carried out a reconstitution experiment by inoculating FMM+12.3 μg/L FdU plates with mixtures of wild-type and HSVtk-expressing conidia in varying ratios. As expected, wild-type (PH-1) conidia germinated on FMM+12.3 μg/L FdU, while the growth of HSVtk-expressing strains was inhibited (see **Supplementary Figure S2**). We could fully suppress germination of HSVtk-expressing conidia by adding FdU to the medium at a final concentration of 12.3 μg/L (50 nM) and we were able to recover the wild-type colonies derived from 10 wild-type spores mixed with 990 HSVtk conidia. We tested the sensitivity of wild-type PH-1 to FdU by plating conidia on plates containing increasing amounts of FdU and found that concentrations of up to 61.5 μg/L had no observable effect on growth rate.

### Transformation Efficiency in *Fusarium graminearum*

In order to measure the transformation efficiency of the dual-selectable fusion markers in comparison to the original resistance genes, we carried out targeted gene disruptions with the three markers. In addition to the plasmids pPS45, pPS48, and pPS51 carrying HSVtk fusions with the selective markers (*nptII, nat1, hph*, respectively), we generated HSVtk-less variants of the same plasmids. The vectors were used to disrupt the *F. graminearum* genes FGSG_02279, FGSG_03278, and FGSG_00348, respectively. We performed three independent transformations with each construct containing the same flanking regions, either with an HSVtk fusion gene or with the corresponding resistance gene alone. Each transformation was carried out using the same amount of DNA (10 μg). This way, we obtained a reasonable number of transformants for each marker and screened them by PCR for in-locus integration. As shown in **Table 1**, the total number of transformants varied widely between the different constructs. Roughly, half of the number of transformants could be obtained with the HSVtk fusions compared to the corresponding HSVtk-less constructs. This can be partly explained by the fact that the HSVtk constructs are ∼30% larger, therefore, the molar amount of DNA used was lower than in the controls. However, the rate of integrations at the desired locus was not negatively affected by the presence of HSVtk. Overall, the use of positive-negative selection markers did not lead to a disadvantage regarding transformation efficiency or homologous integration rate.

**Table 1:** Number of transformants and number of strains with correct integrations obtained during transformation efficiency testing of the HSVtk fusion markers compared to their HSVtk-less (resistance gene only) controls.

	HSVtk-*nptII*	*nptII*	HSVtk-*nat1*	*nat1*	HSVtk-*hph*	*hph*
Number of candidates	35	85	38	39	36	90
Targeted integration	7	9	16	5	6	14
% Correct	20.0%	10.6%	42.1%	12.8%	16.7%	15.6%


*HSVtk-*nptII* and *nptII*: pPS45 and pPS45HL, integration locus: FGSG_02279; HSVtk-*nat1* and *nat1*: pPS48 and pPS48HL, integration locus: FGSG_03278; HSVtk-*hph* and *hph*: pPS51 and pPS51HL, integration locus: FGSG_00348.*

### Activity of Resistance Genes in *E. coli*

Eukaryotic promoters in high copy number plasmids may function also in prokaryotic cells (e.g., Antonucci et al., 1989; Jopcik et al., 2013), which may be useful for construction of disruption constructs in *E. coli* (Güldener et al., 1996). To test whether HSVtk fusion markers confer resistance to *E. coli*, exponential phase cultures of *E. coli* DH10B with HSVtk fusion plasmids were plated onto LB plates containing the antibiotics kanamycin (kan), hygromycin B (hyg), or nourseothricin (nat). The HSVtk-less versions of these plasmids were used as controls for the resistance marker itself. To provide a reference value, each culture was also plated on LB+ampicillin. The functionality of each resistance gene was determined by dividing the number of colonies (CFU) on a kan/hyg/nat selection plate by the CFU count on the reference ampicillin plate of the same strain. *E. coli* DH10B was also plated on each antibiotic concentration to confirm that untransformed *E. coli* was unable to grow on these media.

**FIGURE 2:**
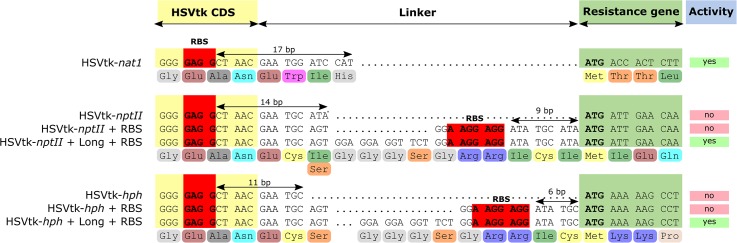
Sequences of the designed linkers separating HSVtk from the resistance genes. Ribosome-binding site (RBS, Shine-Dalgarno sequence) consensus is highlighted in red. Distances between putative RBS and resistance gene start codons are indicated with arrows.

**FIGURE 3:**
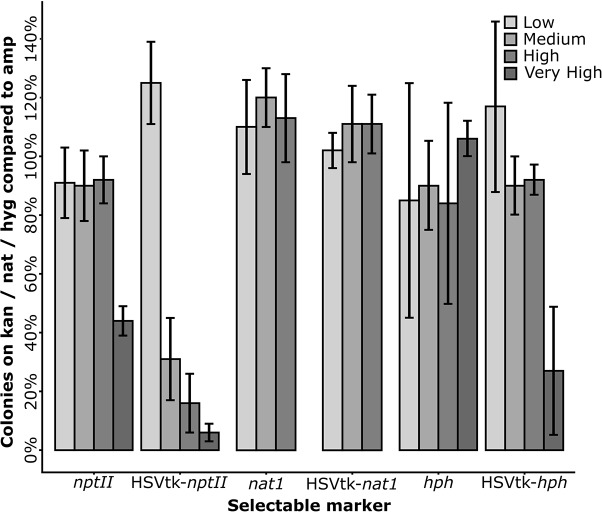
Plating efficiency of *Escherichia coli* transformants on media containing increasing antibiotic concentrations. *E. coli* DH10B transformed with the long linker variants of the HSVtk plasmids pPS45 (HSVtk-*nptII*) and pPS51 (HSVtk-*hph*), the unchanged (short linker) plasmid pPS48 (HSVtk-*nat1*), and the HSVtk-less control plasmids pPS45HL (*nptII*), pPS48HL (*nat1*) and pPS51HL (*hph*) were used. Antibiotic concentrations (low, medium, high, or very high) varied by antibiotic and were as follows: Kanamycin (*nptII*): 15 mg/L; 20 mg/L; 25 mg/L; 30 mg/L. Nourseothricin (*nat1*): 25 mg/L; 37.5 mg/L; 50 mg/L. Hygromycin (*hph*): 32.5 mg/L; 37.5 mg/L; 42.5 mg/L; 50 mg/L. The number of colony forming units (CFU) of each fusion or control construct was divided by the CFU count on the respective ampicillin controls. The average of six repeats per concentration is shown.

Of the three fusion marker plasmids pKT245 (*nptII*), pKT247 (*nat1*), and pKT248 (*hph*), only HSVtk-*nat1* conferred resistance to nourseothricin in *E. coli*, while all of the HSVtk-less controls were able to grow. For the two non-functional HSVtk fusions with *nptII* and *hph*, the linker sequence connecting the *nptII* and *hph* domains to HSVtk was optimized (**Figure 2**). Two new linkers were generated: a short linker (SL) introducing a Shine-Dalgarno sequence upstream of the resistance domain for translation initiation in *E. coli*, and a longer linker (LL) containing both the Shine-Dalgarno sequence and additional bases to achieve better folding of the fusion protein. The shorter version of the two new fusion genes did not confer resistance to *E. coli*. The LL, on the other hand, enabled growth of single colonies, albeit only on plates containing reduced concentrations of hygromycin (37.5 mg/L) and kanamycin (15 mg/L, see **Figure 3**). Despite the reduced antibiotic concentrations in the media, untransformed *E. coli* could not grow, demonstrating that *E. coli* transformants expressing HSVtk fusion constructs can be successfully selected, or screened by transferring candidates to plates with the respective antibiotics.

### Cre-Mediated Excision of the Marker Cassettes in the *PKS12* Gene

To test the reversibility of HSVtk integration, *PKS12* (FGSG_02324) was chosen as a disruption target. *PKS12* encodes a polyketide synthase required for production of the red pigment aurofusarin (Kim et al., 2005; Malz et al., 2005). On solid media, wild-type *Fusarium* mycelia will turn dark red, whereas Δ*pks12* strains remain white. The utility of *PKS12* as a phenotypic marker in *F. graminearum*, e.g., for co-transformation experiments, has been demonstrated previously (Maier et al., 2005).

**FIGURE 4:**
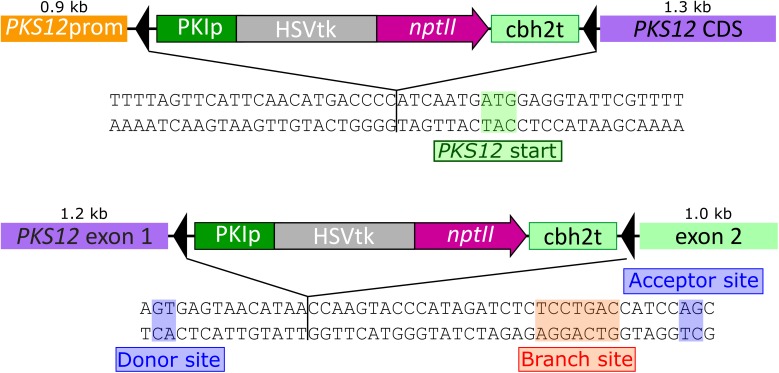
Sequences of the *Fusarium graminearum PKS12* gene highlighting the insertion points of the reversible disruption constructs. Promoter insertion 7 bp upstream of the start codon (pKT257, top) and insertion into the intron between donor site and branch site (pKT258, bottom).

*PKS12* was inactivated using two different approaches, integrating the HSVtk*-nptII* marker either in the promoter upstream of the *PKS12* start codon (pKT257), or into the first 46-bp long intron upstream of the predicted YNCURAY branch point (pKT258). The insertion sites of the markers are shown in **Figure 4**.

**FIGURE 5:**
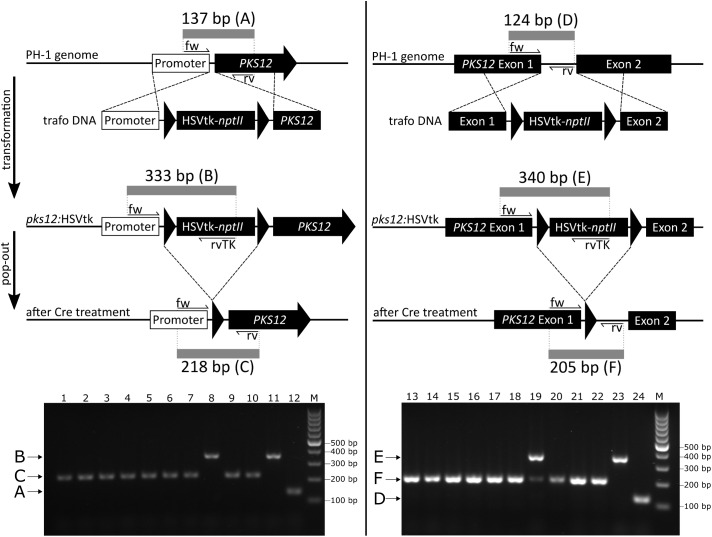
*PKS12* disruption diagram (upper part) and PCR analysis of pop-out candidates from both constructs (lower part). The diagram shows integration of HSVtk-*nptII* marker between promoter and start codon (left) and into the first intron (right) with locations of PCR primers and lengths of the respective PCR products. 1–10, pop-out candidates of promoter integration transformants (pKT257); 11, precursor strain (promoter integration transformant); 12, wild-type control (PH-1); 13–22, pop-out candidates of intron integration transformants (pKT258); 23, precursor strain (intron integration transformant); 24, wild-type control (PH-1). M, GeneRuler^TM^ 100 bp DNA ladder (Thermo Fisher Scientific). Multiplex PCR primers used for intron integration: #4576, #4579, and #4575, for promoter integration: #4577, #4578, and #4575. Trafo DNA, DNA used for transformation.

*Fusarium graminearum* PH-1 was transformed with linearized DNA of either pKT257 or pKT258. Transformants with a disrupted *PKS12* gene exhibited the predicted white mycelium phenotype and correct integration was furthermore confirmed by PCR. White second generation transformants were then subjected to the pop-out protocol by addition of 5 μL purified Cre recombinase to protoplasts, and counterselection using 123 μg/L FdU in the overlay medium (61.5 μg/L final concentration). Cre-free controls resulted in a highly reduced number (about 5%) of FdU-resistant colonies. To check whether the expected pop-out had occurred, 10 candidates of each disruption strategy were screened by PCR. For the two variants using Cre, 9 out of 10 screened candidates were correct, containing only a single remaining *loxP* site (**Figure 5**). PCR of the intron pop-out candidate #9 (lane 19) resulted in two bands. We assume that this is due to an incomplete reaction leading to the presence of both HSVtk-excised and HSVtk^+^ nuclei in a single colony. The presence of the long band solely indicates that the HSVtk cassette is present; it does not account for functionality of the gene. In the case of the observed false-positive candidates, a point mutation in the HSVtk gene might have resulted in the observed FdU resistance. Five strains isolated from the controls without Cre were all false positives (no pop-out), retaining at least the 5′ part of the dual selectable cassette containing the *PKI* promoter (**Supplementary Figure S3**).

**FIGURE 6:**
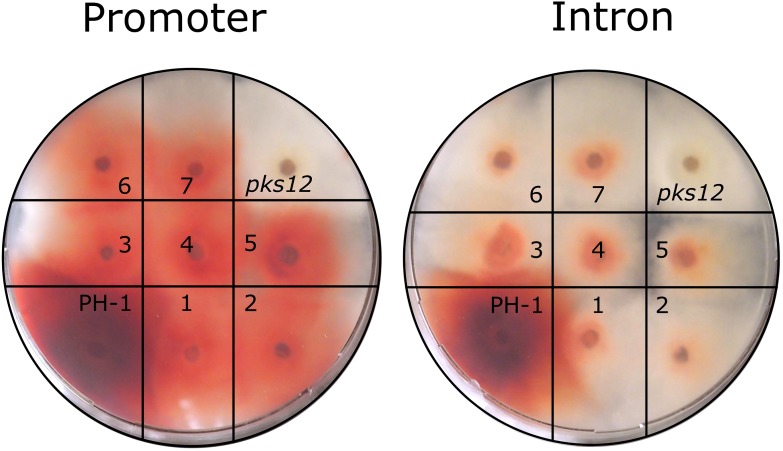
Phenotype testing of *PKS12* disruption and reversion candidates. On each plate, wild-type (PH-1) was placed alongside the *pks12*::HSVtk (*pks12*) mutant (left, promoter integration; right, intron integration) and seven reconstituted, PCR-screened candidates obtained after Cre treatment of the respective strain (1–7). Inoculum: agar plug (1 mm thick and 4 mm in diameter) from 7-day-old cultures, photo taken after 7 days at 20°C.

The reconstituted *PKS12* strains regained the red phenotype during growth on FMM agar (**Figure 6**), demonstrating functional aurofusarin biosynthesis despite the *loxP* insertion into promoter or intron. After the marker pop-out, the red pigmentation in the intron insertion strains was clearly reduced in comparison to the wild type and the promoter insertion strains, and the mycelium turned red later than in the wild type. Since the three different genotypes (wild type, integrated HSVtk, pop-out with remaining *loxP* site) result in clearly distinguishable PCR bands (**Figure 5**), we could show that the observed aurofusarin production was indeed the result of marker removal and was not due to presence of leftover wild-type nuclei.

### Marker Recycling

One application of the marker constructs described above is for multiple rounds of gene deletions using the same marker(s). To confirm that marker recycling indeed works, we consecutively disrupted two genes in the *F. graminearum* PH-1 genome using the HSVtk-*hph* marker. First, the gene FGSG_00348 was deleted by replacing the coding sequence with the HSVtk-*hph* cassette using the vector pPS51. Protoplasts of second-generation transformants (see “Materials and Methods”) were subjected to Cre recombinase treatment and subsequent selection on FdU. Resistant candidates were isolated and screened by PCR. Furthermore, loss of hygromycin resistance was verified by placing mycelium of the pop-out candidates on hygromycin-containing FMM plates. Candidates confirmed by PCR were unable to grow on media containing the antibiotic. The second target gene, FGSG_16976, was then deleted from one second-generation candidate again using the hygromycin marker (pPS50), and resistant candidates were obtained after the transformation. A double knockout strain of both FGSG_00348 and FGSG_16976 was confirmed by PCR (data not shown). This way, we demonstrate that marker recycling is possible, and that several genes can be deleted in one strain using a single selection marker.

To test whether it is possible to remove unselected *loxP*-flanked markers in *F. graminearum*, we integrated an HSVtk-*nptII* marker into a strain already carrying a *loxP*-flanked HSVtk-less *hph* and introduced Cre to protoplasts of the resulting strains. Of 12 PCR-screened FdU-resistant candidates, all had simultaneously lost the HSVtk-*nptII* marker and the second unselected *loxP*-flanked *hph* marker.

### Removal of Marker Cassettes by Homologous Recombination (Marker-Free Allele Swap)

The *F. graminearum TRI8* gene (FGSG_03532) encodes a trichothecene-3-O-esterase, which preferentially cleaves off the 3-acetyl group of the trichothecene mycotoxin 3,15-diacetyl-deoxynivalenol (3,15-diADON) to produce 15-ADON. However, certain isolates of *F. graminearum* were found to possess an allele of *TRI8* with a preference to cleave the 15-acetyl group instead, mainly producing 3-ADON instead of 15-ADON (Alexander et al., 2011). To generate a *F. graminearum* PH-1-based strain producing 3-ADON instead of 15-ADON, we used the HSVtk fusion vectors and generated a marker-free *TRI8* allele swap strain in a two-step transformation. In the first step, we integrated an HSVtk-*nat1* cassette without *loxP* sites in place of the native *TRI8* gene. In the second step, we replaced the HSVtk marker with the 3-ADON *TRI8* allele flanked by regions of about 500 bases on each side. After 3–5 days, transformants emerged from the regeneration agar and were transferred to FMM+FdU plates. FdU-resistant colonies were screened (*n* = 5) for the desired double crossover event removing the HSVtk locus by homologous recombination, which allowed the detection of the desired event in one candidate.

Using both Cre-catalyzed and homology-based marker removal methods, some of the transformants emerging from the FdU containing agar (123 μg/L) were incapable of growing on minimal medium containing 12.3 μg/L FdU. The higher escape rate on rich medium as compared to minimal medium is possibly due to dilution of the toxic base analog (250 nM in regeneration medium) by nucleotides present in 1 g/L yeast extract (5′ UMP: ∼150 μM according to Neubauer et al., 2012). Although the fraction approaches 50% in some cases, this does not pose a problem, since such candidates are completely inhibited on FdU-containing minimal media and are removed from the candidate pool without further screening effort.

## Discussion

We generated a series of transformation vectors and developed a method for efficient marker recycling in *Fusarium* based on the Cre*-loxP* system. To prevent problems associated with baseline Cre expression due to leaky promoters, our marker removal protocol uses direct introduction of Cre recombinase. We had previously used a self-excising cassette based on inducible Cre under the *Trichoderma* xylanase promoter, as described by Steiger et al. (2011), but frequently encountered problems with transformants that were no longer able to excise the cassette after two rounds of purification by sporulation. We observed that in some cases C-terminal truncations of the Cre gene occurred already in *E. coli*, probably due to background Cre expression. Furthermore, in *F. graminearum*, leaky expression on the complex regeneration medium or sporulation medium combined with selection for the antibiotic resistance marker resulted in transformants that were later unable to excise the cassette.

The commonly used counterselection system based on amdS and fluoroacetamide did not work in our hands for *F. graminearum*, while counterselection based on HSVtk and FdU had a low rate of escapes. Marker removal with purified Cre protein showed to be robust. We found that direct addition of Cre recombinase to *F. graminearum* protoplasts does not require a DNA carrier to be added to the reaction mixture, in contrast to the original report (Mizutani et al., 2012) working with *Aspergillus oryzae*. A possible reason for this might be that our homemade Cre recombinase preparation has a higher activity than commercial preparations. Alternatively, *F. graminearum* protoplasts might be more efficient in protein uptake than those of *A. oryzae*. A possible alternative method for introduction of Cre by anastomosis has been reported for *Cryphonectria* (Zhang et al., 2013).

Based on the data from the Cre-free controls of the *PKS12* reconstitution experiments we conclude that the spontaneous recombination frequency at the direct repeat *loxP* sites is very low. The few FdU-resistant pop-out candidates obtained without Cre were false positives, which had not deleted the cassette but seemingly truncated the HSVtk gene (see **Supplementary Figure S3**).

The HSVtk fusion plasmids, as shown in **Figure 1**, were optimized as backbones for digestion/ligation cloning by introduction of multiple cloning sites up- and downstream of the promoter and terminator regions. The resistance cassettes can also be isolated via restriction digest or PCR for Gibson assembly reactions, which allows for rapid generation of a series of consecutive disruption vectors. So far, we have used the HSVtk dual-selectable markers in several experiments requiring marker-free transformations, most notably to consecutively inactivate seven members of a gene family in *F. graminearum* PH-1 (Svoboda et al., in preparation). Yet, if used for multiple sequential knockouts, the presence of multiple *loxP* sites in the genome comes with the risk of unwanted chromosomal rearrangements or deletions. Alternatively, the HSVtk-fusion vectors lacking *loxP* sites could be used in a single transformation pop-in pop-out procedure as described recently in *Magnaporthe oryzae* for four sequential knockouts of genes encoding nep-1 like proteins (Fang et al., 2017).

In our *PKS12* deletion experiment, we could show that introducing a *loxP* site into an intron can negatively influence gene expression, while integration between the promoter and the start codon seems to have only a slight effect (**Figure 6**). The strongly diminished red coloration in the *PKS12* intron pop-out strains might be explained by the strong secondary structure of the *loxP* site being too close to the branch site, thus reducing splicing efficiency. Reversible integration of a sequence of interest at the *loxP* site in the *PKS12* promoter that is accompanied by a visually detectable phenotypic change could be developed into an interesting genetic tool, similar to the *ADE2* red/white colony system frequently employed in *Saccharomyces cerevisiae* (De la Cruz et al., 1998).

Homology-based recombination for seamless allele swaps or deletions is not as efficient as Cre*-loxP* mediated marker removal, and the number of transformants obtained is generally lower, depending mainly on integration locus. We have successfully employed this method to obtain near isogenic PH-1 derived strains differing in the chemotype, producing either 3- or 15-ADON *in vitro* as confirmed by chemical analysis.

## Conclusion

In summary, we have constructed fusion genes allowing positive and negative selection in *Fusarium* and most likely in other related ascomycetes. These fusion genes are much smaller than cassettes containing the positive and negative selectable marker driven by separate promoters and terminators. Testing of the transformation efficiency and the rate of homologous recombination showed that the vectors can be used without a large penalty in forward selection for gene disruption. The HSVtk fusion genes flanked by *loxP* sites can be efficiently popped out using purified Cre protein added to protoplasts, allowing marker recycling and opening the possibility for repeated gene disruption in one strain. The negative selection with HSVtk lacking flanking *loxP* sites is strong enough to directly use the counterselection for introduction of allelic changes, allowing to study the effects in an otherwise near isogenic background.

## Data Availability Statement

The plasmid sequences generated for this study can be found in the GenBank database under the following accession numbers:

pKT300 MH286798
pKT301 MH286799
pKT302 MH286800
pKT303 MH286801
pKT304 MH286802
pKT305 MH286803
pKT311 MH286804
pKT312 MH286805
pKT313 MH286806

## Author Contributions

KT and PS constructed the plasmids. KT performed the experiments. HM purified and tested Cre recombinase. GW and GA conceived the concept. GW designed the constructs and supervised experimental work. KT, GW, and GA wrote the paper and all authors amended and corrected the paper.

## Conflict of Interest Statement

The authors declare that the research was conducted in the absence of any commercial or financial relationships that could be construed as a potential conflict of interest.

## Footnotes

1 http://tmcalculator.neb.com
