# Supplementary material for: New Plasmids for Fusarium Transformation Allowing Positive-Negative Selection and Efficient Cre-loxP Mediated Marker Recycling
Source: Front Microbiol. 2018 Sep 11;9:1954. doi: 10.3389/fmicb.2018.01954 (PMC6143793; doi:10.3389/fmicb.2018.01954)
Supplement: Supplementary file 1 [file Data_Sheet_1.pdf]

Supplementary Material

New plasmids for *Fusarium* transformation allowing positive-negative selection and efficient Cre-*loxP* mediated marker recycling

Krisztian Twaruschek[1], Pia Spörhase[1,2], Herbert Michlmayr[1], Gerlinde Wiesenberger[1*], Gerhard Adam[1]

[1]Department of Applied Genetics and Cell Biology, University of Natural Resources and Life Sciences, Vienna (BOKU), Konrad Lorenz Str. 24, A-3430 Tulln, Austria

*** Correspondence:**
Corresponding Author
gerlinde.wiesenberger@boku.ac.at

[2]Present address: Maquet Cardiopulmonary GmbH, 72379 Hechingen, Germany

1 Supplementary Figures and Tables

1.1 Supplementary Tables

Supplementary Table 1: Screening PCRs for verification of *Fusarium* transformant genotype. Cycler parameters: 30 sec denaturation, 30 sec annealing, 1 min/kb elongation at 72°C, 30 cycles. (*): band does not indicate wild-type, but precursor strain (HSVtk integration) instead. Trafo band, band for correct transformant; T_m, annealing temperature.

target gene	vector	fw PH-1	rv PH-1	rv vector	trafo band [bp]	wild-type band [bp]	pop-out band [bp]	T_m [°C]
**FGSG_02279 (5')**	pPS45 5'	2802	2803	3579	1395	995	N/A	63
**FGSG_03278 (5')**	pPS48 5'	2928	2930	3952	1158	832	N/A	63
**FGSG_00348 (5')**	pPS51 5'	3992	3993	3580	1053	869	N/A	63
**FGSG_16976 (5‘)**	pPS50 5‘	3988	3989	3580	1198	960	N/A	55
***PKS12* promoter**	pKT257	4579	4576	4575	333	137	218	63
***PKS12* intron**	pKT258	4577	4578	4575	340	124	205	63
***FgTRI8* swap**	pKT299	4142	4765	3462	905	1215*	N/A	55

Supplementary Table 2: A list of primers used in this study.

Cloning primers		Fragment / cloning technique
**2783**	TAATGGCCGCATAGGCCAGGTTCTAACTCGTAACGTT	FGSG_02279 5' UTR fw for digestion/ligation
**2784**	TTAACTAGTGCTGGATAAGACAATAGGTA	FGSG_02279 5' UTR rv for digestion/ligation
**2785**	ATTGTCGACATTATCAGTTTGTAAAAATATGA	FGSG_02279 3' UTR fw for digestion/ligation
**2786**	TATCGTACGCGACGATCCAGATTCATC	FGSG_02279 3' UTR rv for digestion/ligation
**2827**	TAATGGCCGCATAGGCCTCTCGAGTAGAGTCTTTGCTA	FGSG_03278 5' UTR fw for digestion/ligation
**2828**	TATACTAGTCTGGAGCGTGATCAAGATGA	FGSG_03278 5' UTR rv for digestion/ligation
**2829**	CAAGTCGACCCTCAACCCCGTCAATGGA	FGSG_03278 3' UTR fw for digestion/ligation
**2830**	TATAAGCTTCGACTAATAATTGTATATTGAGAT	FGSG_03278 3' UTR rv for digestion/ligation
**3099**	ATATCTAGATGGCTTCGTACCCCTGCCAT	HSVtk fw for digestion/ligation
**3100**	ATAGGTACCTCAGTTAGCCTCCCCCATCTCCC	HSVtk rv for digestion/ligation
**3157**	TGTTCAATCATTATGCATTCGTTAGCCTCCCCCATCTCC	HSVtk rv fusion for fusion PCR
**3158**	ATAGGTACCTCAGAAGAACTCGTCAAGAAGG	nptII rv for digestion/ligation
**3159**	GAATGCATAATGATTGAACAAGATGGATTGCAC	nptII fw for fusion PCR
**3559**	TATGCGGCATCAGAGCAGATTGTACTGAGAGTGCACC AGACTTTTCTCAGGCCTCGCTG	FGSG_03532 5' UTR fw for assembly
**3561**	TATTCATCACAACCTCGTCTCCCTCCTTTGGGTTGAGCT CCTCGAGTTTCATTTCTTGACATCTCTGGCCAT	FGSG_03532 3' UTR fw for assembly
**3573**	ACAGCTGGATAAGGGACCGGCCGCTAGTCTCACCGTT ATCGGATCCACTGAGTGAGTGAACGAATGAATGAC	FGSG_03532 5' UTR rv for assembly
**3574**	AGCTATGACCATGATTACGCCAAGCTTGCATGCCTGCA GGCATATGACGATAACTACAGCCTC	FGSG_03532 3' UTR rv for assembly
**3844**	ATAGAATGCATGAAAAAGCCTGAACTCACCG	hph fw for digestion/ligation
**3845**	ATAGGTACCCTATTCCTTTGCCCTCGGACG	hph rv for digestion/ligation
**3942**	ACTATGCGGCATCAGAGCAGATTGTACTGAGAGTGCA CCATTCGCTTGGCCTTGCTCCACT	FGSG_16976 5' UTR fw for assembly
**3943**	CGTATAGCATACATTATACGAAGTTATATTAAGGGTTG TCGAGATGAGATAATGTCTGGGTACTC	FGSG_16976 5' UTR rv for assembly
**3944**	AATGTATGCTATACGAAGTTATTAGGTGATATCAGATC CAGTGTGCCGTGTGTGGGAGAT	FGSG_16976 3' UTR fw for assembly
**3945**	CTATGACCATGATTACGCCAAGCTTGCATGCCTGCAGG TCGAGGTGAGGCACATGGTCGGGT	FGSG_16976 3' UTR rv for assembly

**3946**	ACTATGCGGCATCAGAGCAGATTGTACTGAGAGTGCA CCACCTCCCTCATTGCCACCACC	FGSG_00348 5' UTR fw for assembly
**3947**	CGTATAGCATACATTATACGAAGTTATATTAAGGGTTG TCGAGGTGACAGATGAGTCTGAAGAAG	FGSG_00348 5' UTR rv for assembly
**3948**	TGTATGCTATACGAAGTTATTAGGTGATATCAGATCCA CCAGAACAATGGCGTTGATGCA	FGSG_00348 3' UTR fw for assembly
**3949**	GACCATGATTACGCCAAGCTTGCATGCCTGCAGGTCGA TTTGCCACTACCGCAAGCATCT	FGSG_00348 3' UTR rv for assembly
**3966**	TACGAAGTTATATTAAGGGTTGTCGACCTGCAGCGTAC GATTATGTTACTCACTGAATGAAACTG	FgPKS12 (intron) 5' UTR rv for assembly
**3967**	AATGTATGCTATACGAAGTTATTAGGTGATATCAGATC CACCAAGTACCCATAGATCTCTC	FgPKS12 (intron) 3' UTR fw for assembly
**3969**	ACTATGCGGCATCAGAGCAGATTGTACTGAGAGTGCA CCATGATCAGGCGCAACCTTTTC	FgPKS12 (promoter) 5'&3' UTR fw for assembly
**3970**	TACGAAGTTATATTAAGGGTTGTCGACCTGCAGCGTAC GAGGGGTCATGTTGAATGAACT	FgPKS12 (promoter) 5' UTR rv for assembly
**3971**	AATGTATGCTATACGAAGTTATTAGGTGATATCAGATC CAATCAATGATGGAGGTATTCGT	FgPKS12 (promoter) 3' UTR fw for assembly
**3972**	AGCTATGACCATGATTACGCCAAGCTTGCATGCCTGCA GGGAATTCACCCAAGTCAGCTG	FgPKS12 (promoter) 5'&3' UTR rv for assembly
**4228**	GTGGAAGGAGGATATGCA	Short linker oligo fw
**4229**	TATCCTCCTTCCACTGCA	Short linker oligo rv
**4230**	GTGGAGGAGGTTCTGGAAGGAGGATATGCA	Long linker oligo fw
**4231**	TATCCTCCTTCCAGAACCTCCTCCACTGCA	Long linker oligo rv
**4769**	CATTCGTTCACTCACTCAGTATGGTTCTCGATCGTTTGT TGTT	3-ADON TRI8 fw for assembly
**4770**	AGAAATGAAATCACAACTCTCGTGTTACGTCTC	3-ADON TRI8 rv for assembly
**4771**	AGAGTTGTGATTTCATTTCTTGACATCTCTGGCC	3-ADON TRI8 3'UTR fw for assembly

Screening primers for *Fusarium* genomic integration		Binding site / orientation
**2802**	CGAAACTTGTCGGCTGTGG	FGSG_02279 wildtype fw
**2803**	GGTGGGAGCTTGATCTTGG	FGSG_02279 wildtype rv
**2928**	TGGGGAGTCATGATGCGATA	FGSG_03278 wildtype fw
**2930**	GAACATAGTACGCGACAAAGA	FGSG_03278 wildtype rv
**3462**	ATGGACCAAAGGCAGACAAGG	FGSG_03532 KO vector rv
**3579**	GTAGACCGCAAATGAGCAAC	FGSG_02279 KO vector rv
**3580**	GCCACAGCAGCCACGACA	FGSG_00348 KO vector rv
**3952**	ATCTAAAGATCGATTCGGCAG	FGSG_03278 KO vector rv
**3988**	GATCATTATCAGGAAGTCTCG	FGSG_16976 wildtype fw
**3989**	CGCCTCGATCACCTCTGTC	FGSG_16976 wildtype rv
**3992**	AGCTGCTTAGGTCTCGTGAT	FGSG_00348 wildtype fw
**3993**	ATCGGGCTGGGGACTGTG	FGSG_00348 wildtype rv
**4142**	GCTGGTGCTGGAAAAGATTC	FGSG_03532 wildtype fw
**4575**	TCTGTGAGCCAACCAAGGGC	PKS12 HSVtk vector rv
**4576**	TTTAACAAAATGTGTCAAGTAAGGACTGT	PKS12 promoter insertion wildtype rv
**4577**	ACCATCTTTGCAGTTTCATTCAGTGAG	PKS12 intron insertion wildtype fw
**4578**	GTCCAATAATGCGACTGTTCTCCG	PKS12 intron insertion wildtype rv
**4579**	ACTCTTTTAGTTCATTCAACATGACCCC	PKS12 promoter insertion wildtype fw
**4765**	AGAATGTTGTAAGAAGCAGA	3-ADON TRI8 mid rv

Supplementary Table 3: List of plasmids used in this study

Name	Genotype	Purpose	Reference
pRLMex30	*ampR, PKIprom-hph-CBH2term, pUC ori*	Fungal transformation vector with hph marker	*Mach et al., 1994*
pAB86	*ampR, PKIprom-XbaI-KpnI-gpdA-hph-CBH2term*	Fungal overexpression vector (PKIprom) with hph resistance	*This study*
pCGS966	*ampR, HSVtk*	Yeast vector with counterselectable HSV thymidine kinase gene	*Smith et al., 1990*
pKT235	*ampR, PKIprom-nptII-CBH2term, pUC ori*	HSVTK overexpression test construct	*This study*
pKT241	*ampR, PKIprom-HSVtk-nptII-gpdA-hph-CBH2term, pUC ori*	HSVTK-nptII overexpression test construct	*This study*
pTS101	*ampR, loxP-PKIprom-hph-CBH2term-loxP, pUC ori*	loxP-flanked resistance cassette from pRLMex30	*This study*
pKT244	*ampR, loxP-PKIprom-HSVtk-nptII-gpdA-hph-CBH2term-loxP, pUC ori*	loxP-flanked HSVtk-nptII resistance cassette with hph marker	*This study*
pKT245	*ampR, loxP-PKIprom-HSVtk-nptII-loxP, pUC ori*	loxP-flanked HSVtk-nptII resistance cassette	*This study*
pKT246	*ampR, loxP-PKIprom-HSVtk-nat1-gpdA-hph-CBH2term-loxP, pUC ori*	loxP-flanked HSVtk-nat1 resistance cassette with hph marker	*This study*
pKT247	*ampR, loxP-PKIprom-HSVtk-nat1-loxP, pUC ori*	loxP-flanked HSVtk-nat1 resistance cassette	*This study*
pKT248	*ampR, loxP-PKIprom-HSVtk-hph-loxP, pUC ori*	loxP-flanked HSVtk-hph resistance cassette	*This study*
pKT292	*ampR, loxP-PKIprom-HSVtk-longlinker-nptII-loxP, pUC ori*	loxP-flanked HSVtk-nptII resistance cassette, long linker	*This study*
pKT293	*ampR, loxP-PKIprom-HSVtk-longlinker-hph-loxP, pUC ori*	loxP-flanked HSVtk-hph resistance cassette, long linker	*This study*
pKT290	*ampR, MCS, pUC ori*	Empty pKT245 backbone with ligated polylinker, pKT3XX precursor	*This study*
pKT300	*ampR, MCS1-loxP-PKIprom-HSVtk-longlinker-nptII-loxP-MCS2, pUC ori*	loxP-flanked HSVtk-nptII long linker resistance cassette with two MCS	*This study*
pKT301	*ampR, MCS1-loxP-PKIprom-HSVtk-nat1-loxP-MCS2, pUC ori*	loxP-flanked HSVtk-nat1 resistance cassette with two MCS	*This study*
pKT302	*ampR, MCS1-loxP-PKIprom-HSVtk-longlinker-hph-loxP-MCS2, pUC ori*	loxP-flanked HSVtk-hph long linker resistance cassette with two MCS	*This study*
pKT303	*ampR, MCS1-PKIprom-HSVtk-longlinker-nptII-MCS2, pUC ori*	HSVtk-nptII long linker resistance cassette with two MCS	*This study*
pKT304	*ampR, MCS1-PKIprom-HSVtk-nat1-MCS2, pUC ori*	HSVtk-nat1 resistance cassette with two MCS	*This study*
pKT305	*ampR, MCS1-PKIprom-HSVtk-longlinker-hph-MCS2, pUC ori*	HSVtk-hph long linker resistance cassette with two MCS	*This study*
pKT311	*ampR, MCS2-PKIprom-HSVtk-longlinker-nptII-MCS1, pUC ori*	Reversed HSVtk-nptII long linker resistance cassette with two MCS	*This study*
pKT312	*ampR, MCS2-PKIprom-HSVtk-nat1-MCS1, pUC ori*	Reversed HSVtk-nat1 resistance cassette with two MCS	*This study*
pKT313	*ampR, MCS2-PKIprom-HSVtk-longlinker-hph-MCS1, pUC ori*	Reversed HSVtk-hph long linker resistance cassette with two MCS	*This study*
pET21a(+)	*ampR, T7 prom-lac operator-T7 tag-MCS-T7 term, pUC ori*	Standard expression plasmid	*Novagen inc.*
Cre-pET21a(+)	*ampR, T7 prom-lac operator-Cre-His-tag-T7 term, pUC ori*	Cre expression construct	*This study*
pMS-HALS	*ampR, loxP-amdS-hph-loxP, sacB, pUC ori*	cloning of pASB2 (amdS/loxP cassette)	*Steiger et al, 2011*
pUni51	*kanR, loxP, R6Kγ ori* – requires a Pir1 host strain	cloning of pASB2 (backbone with kanR marker)	*AccNo. AY260846.1*

pASB2	*kanR, loxP-amdS-hph-loxP, R6Kγ ori* – requires a Pir1 host strain	pASB43 insert (loxP-flanked amdS/hph) in non-ampR plasmid	*This study*
pUG6	*ampR, loxP-kanR-loxP, pUC ori*	to obtain pASB43 backbone	*Gueldener et al, 2002*
pUG6woKAN	*ampR, loxP, pUC ori*	pASB43 backbone	*This study*
pASB43	*ampR, loxP-amdS-hph-loxP, pUC ori*	Fungal transformation vector with amdS/hph marker and ampR	*This study*
pHE31	*ampR, loxP-amdS-hph-loxP, pUC ori, FGSG_02279 5' UTR*	Precursor of pHE49	*This study*
pHE49	*ampR, loxP-amdS-hph-loxP, pUC ori, FGSG_02279 5'* and *3' UTRs*	FGSG_02279 disruption construct, pASB43 backbone	*This study*
pPS45	*ampR, loxP-HSVtk-nptII-loxP, pUC ori, FGSG_02279 5'* and *3' UTRs*	FGSG_02279 disruption construct, counterselectable, pHE49 backbone	*This study*
pPS45HL	*ampR, HSVtk-nptII, pUC ori, FGSG_02279 5'* and *3' UTRs*	nptII transformation efficiency testing construct & control	*This study*
pPS19	*ampR, loxP-amdS-hph-loxP, pUC ori, FGSG_03278 5'* and *3' UTRs*	FGSG_03278 disruption construct, pASB43 backbone	*This study*
pPS48	*ampR, loxP-HSVtk-nat1-loxP, pUC ori, FGSG_03278 5'* and *3' UTRs*	nat1 transformation efficiency testing construct & control	*This study*
pPS48HL	*ampR, loxP-nat1-loxP, pUC ori, FGSG_03278 5'* and *3' UTRs*	FGSG_03278 disruption construct, counterselectable, pPS19 backbone	*This study*
pPS51	*ampR, loxP-HSVtk-hph-loxP, pUC ori, FGSG_00348 5'* and *3' UTRs*	FGSG_00348 disruption construct, counterselectable, pKT248 backbone	*This study*
pPS51HL	*ampR, loxP-hph-loxP, pUC ori, FGSG_00348 5'* and *3' UTRs*	hph transformation efficiency testing construct & control	*This study*
pKT257	*ampR, loxP-HSVtk-nptII-loxP, pUC ori, FGSG_02324* promoter integration	reversible FGSG_02324 promoter integration construct, counterselectable	*This study*
pKT258	*ampR, loxP-HSVtk-nptII-loxP, pUC ori, FGSG_02324* intron integration	reversible FGSG_02324 intron integration construct, counterselectable	*This study*
pPS50	*ampR, loxP-HSVtk-hph-loxP, pUC ori, FGSG_16976 5'* and *3' UTRs*	FGSG_16976 disruption construct, counterselectable, pKT248 backbone	*This study*
pKT249	*ampR, HSVtk-nat1, pUC ori, FGSG_03532 5'* and *3' UTRs*	FGSG_03532 deletion construct, counterselectable	*This study*
pKT299	*ampR, FGSG_3532 5' UTR-3ADON TRI8-3' UTR, pUC ori*	FGSG_03532 allele swap construct (3-ADON allele)	*This study*

Supplementary Table 4: List of flanking homologous regions amplified by PCR for generation of disruption constructs

PCR product	Gene	Fw primer	Rv primer	Amplicon size	Position of first/last nucleotide relative to start/stop codon
**pPS45 5’ UTR**	FGSG_02279	2783	2784	500	-500
**pPS45 3’ UTR**	FGSG_02279	2785	2786	500	+500
**pPS48 5’ UTR**	FGSG_03278	2827	2828	571	-576
**pPS48 3’ UTR**	FGSG_03278	2829	2830	519	+31
**pPS51 5’ UTR**	FGSG_00348	3946	3947	596	-596
**pPS51 3’ UTR**	FGSG_00348	3948	3949	579	+579
**pKT257 5’ UTR**	FGSG_02324	3969	3970	885	-892
**pKT257 3’ UTR**	FGSG_02324	3971	3972	1289	-5181
**pKT258 5’ UTR**	FGSG_02324	3969	3966	1180	+288
**pKT258 3’ UTR**	FGSG_02324	3967	3972	995	-5181
**pPS50 5’ UTR**	FGSG_16976	3942	3943	531	-531
**pPS50 3’ UTR**	FGSG_16976	3944	3945	605	+605
**pKT249 5’ UTR**	FGSG_03532	3559	3573	532	-532
**pKT249 3’ UTR**	FGSG_03532	3561	3574	819	+819
**pKT299 5’ UTR**	FGSG_03532	3559	3573	532	-532
**pKT299 3’ UTR**	FGSG_03532	4771	3574	819	+819

1.2 Supplementary Figures

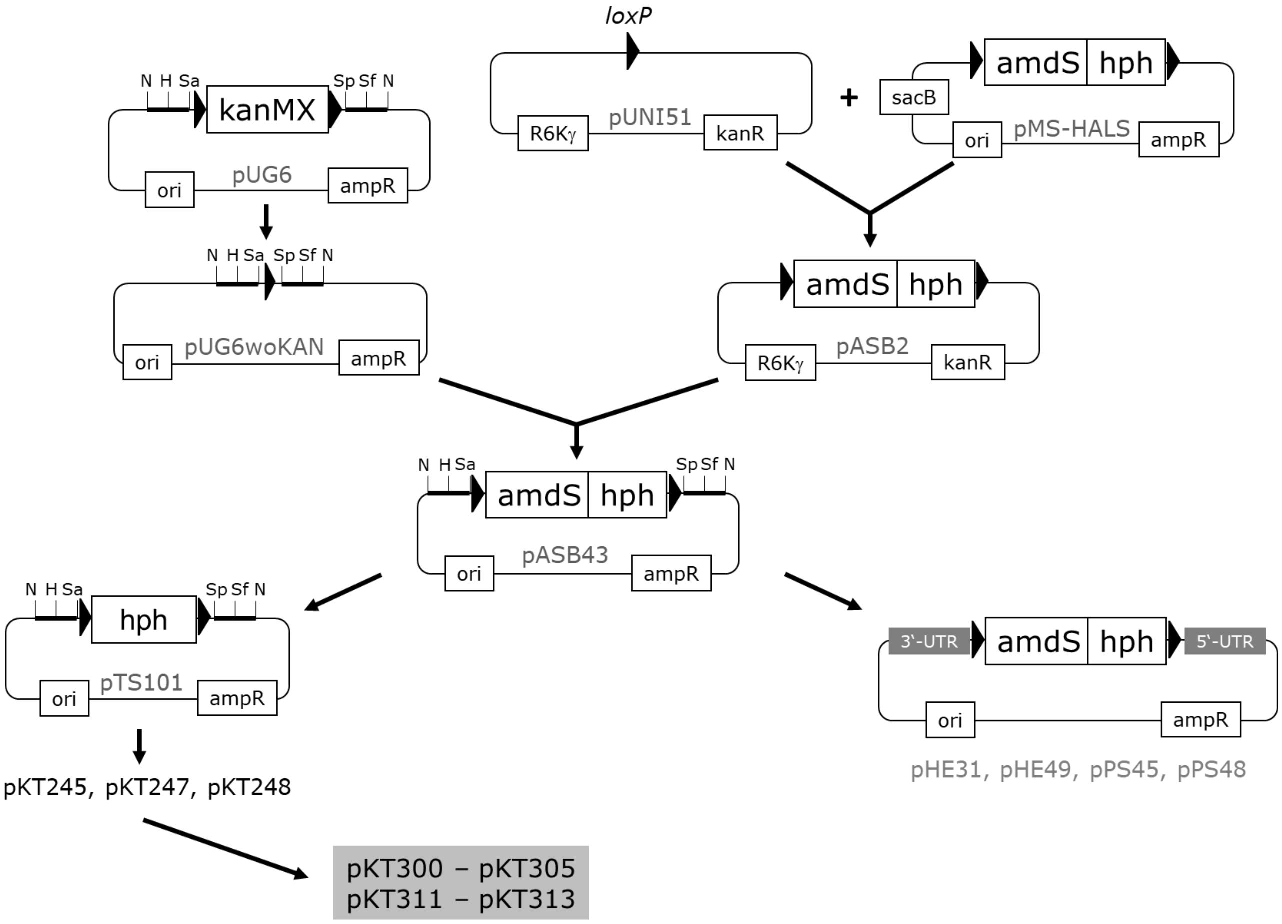

Supplementary Figure 1: Construction scheme of plasmids described in this paper. Recognition sites for restriction enzymes: N, NotI; H, HindIII; Sa, SalI; Sp, SpeI; Sf, SfiI. pKT300 to pKT305 and pKT311, pKT312 and pKT313 are constructs with modified polylinker (see Figure 1 in main body).

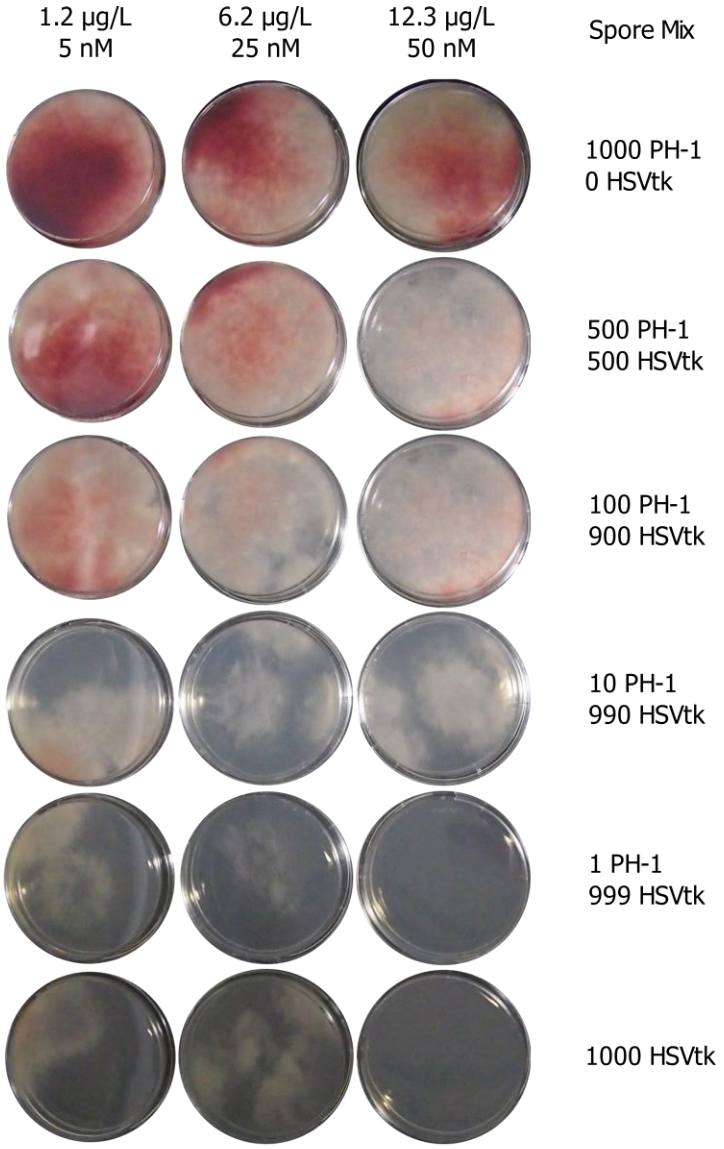

Supplementary Figure 2: Reconstitution experiment on FMM agar containing 5, 25 and 50 nM FdU. Conidia of a thymidine kinase expressing strain (*HSVTK*) were mixed with wild-type (PH-1) spores in as indicated and plated (1000 spores/94mm agar plate). Photo taken after 7 days at 20°C.

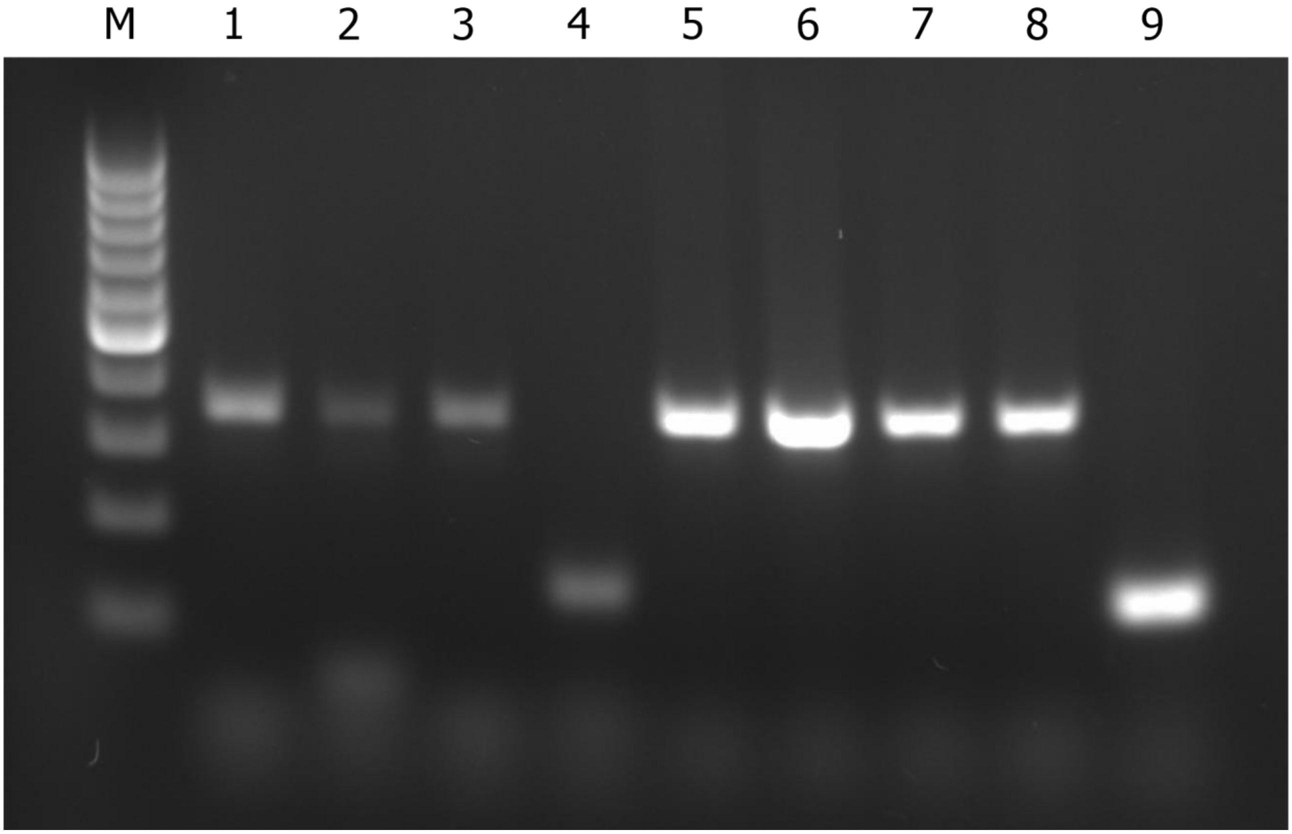

Supplementary Figure 3: PCR screening of five FdU resistant *pks12*Δ candidates obtained without Cre treatment. M, Thermo Gene Ruler™ 1 kb ladder, 1-2, promoter popout candidates (pKT258); 3, pks12 promoter::HSVtk; 4, wild type; 5-7, intron popout candidates (pKT257); 8, pks12 intron::HSVtk; 9, wild type.
